# Involvement of DNA-PKcs in the IL-6 and IL-12 Response to CpG-ODN Is Mediated by Its Interaction with TRAF6 in Dendritic Cells

**DOI:** 10.1371/journal.pone.0058072

**Published:** 2013-03-22

**Authors:** Chi Ma, Marianna Muranyi, Catherine H. Chu, Jianhua Zhang, Wen-Ming Chu

**Affiliations:** 1 Cancer Biology Program, University of Hawaii Cancer Center, University of Hawaii, Honolulu, Hawaii, United States of America; 2 Department of Pediatrics, Shanghai 6^th^ People’s Hospital, Shanghai Jiaotong University, Shanghai, People’s Republic of China; Uppsala University, Sweden

## Abstract

CpG-ODN stimulates dendritic cells (DCs) to produce cytokines, which are important for pathogenesis of autoimmune disorders and vaccine strategy for cancer. CpG-ODN activates the TLR9/MyD88/TRAF6 cascade leading to activation of IKK-NF-κB and JNK, which are critical for production of pro-inflammatory cytokines. However, whether other molecules are involved in activation of CpG-ODN signaling is still not clear. Here we report that the catalytic subunit of DNA-dependent protein kinase (DNA-PKcs) is involved in this activation process. DNA-PKcs-deficient DCs exhibited a defect in the IL-6 and IL-12 response to CpG-ODN in a dose- and time- dependent manner. Loss of DNA-PKcs impaired phosphorylation of IKK, IκBα, NF-κB and JNK in response to CpG-ODN. Interestingly, CpG-ODN was able to bind DNA-PKcs and induce its association and co-localization with TRAF6 in the absence of TLR9. Our data suggest that DNA-PKcs is a player in CpG-ODN signaling and may explain why DNA-PKcs is implicated in the pathogenic process of autoimmune disease.

## Introduction

Oligodeoxynucleotides containing CpG motif (CpG-ODNs) are powerful activators of the innate immune system. There are three types of CpG-ODNs, CpG-A, CpG-B and CpG-C, which is the mixture of CpG-A and CpG-B. CpG-A prefers activating plasmacytoid DCs (pDCs), whereas CpG-B (here we call it CpG-ODN) efficiently activates B cells, conventional dendritic cells (cDCs) and macrophages [1]. CpG-ODN strongly activates cDCs to produce pro-inflammatory IL-6 and IL-12, which is critical for the Th1 response. Thus, CpG-ODN has widely been used as an adjuvant for vaccine strategy for infectious disease and cancer. It is known that CpG-ODN activates its receptor Toll-like receptor 9 (TLR9), which in turn recruits the adaptor protein myeloid differentiation factor 88 (MyD88) and interleukin 1 receptor-associated kinase 4 (IRAK4) leading to phosphorylation and activation of IRAK4 [2]. Activated IRAK4 associates with TNF receptor-associated factor 6 (TRAF6) and triggers its ubiquitination and the subsequent activation of TGF beta-activated kinase 1 (TAK1). Activated TAK1 phosphorylates IκBα kinases (IKKα and IKKβ), which in turn phosphorylate IκBα resulting in NF-κB activation. TAK1 also activates mitogen-activated protein kinases (MAPKs) such as JNK leading to activation of activating protein 1 (AP-1). Both AP1 and NF-κB are critical transcription factors for expression of pro-inflammatory cytokines IL-6 and IL-12.

In addition to above relay molecules, other proteins are also suggested to be transducers in CpG-ODN signaling. One of them is the catalytic subunit of DNA-dependent protein kinase (DNA-PKcs), which is in both the cytoplasm and nucleus of mouse cells [3]. DNA-PKcs is an essential component of double-stranded DNA break repair complex and is vital for B and T cell development [4]. A high level of anti-DNA-PKcs autoantibody is frequently detected in serum of patients with polymyositis, scleroderma, systemic lupus erythematosus, and mixed connective tissue disease [5]. Although the molecular mechanism underlying the implication of DNA-PKcs in the autoimmune pathogenesis is unclear, recent studies suggest that it regulates activation of Akt and innate immune responses. For example, DNA-PKcs can be activated by CpG-ODN *in vitro* and in macrophages where it triggers activation of Akt [3], which has recently been reported to regulate the type I IFN response to CpG-ODN [6]. Interestingly, chloroquine, which abolishes TLR9 activation by CpG-ODN, had no apparent inhibitory effect on Akt activation by CpG-ODN in THP1 macrophages [7]. Moreover, S6 kinase (S6K), a downstream event of Akt, was found to be critical for CpG-ODN-induced association of TLR9 with MyD88 [6], indicating that Akt and S6K might act upstream of TLR9 in CpG-ODN signaling.

A new study using severe combined immune deficiency (SCID) mice, which harbor a point mutation in DNA-PKcs gene [4], suggested that IL-10 expression in SCID macrophages in response to CpG-ODN was almost abolished, whereas the IL-12 response was largely enhanced [8]. However, SCID cells still contain DNA-PKcs at a certain level and have leaking DNA-PKcs activity [4], and therefore the phenotype observed in SCID cells may not be the same as that in DNA-PKcs knockout cells. Moreover, the cytoplasmic role of DNA-PKcs in DCs is still unclear and the relationship between DNA-PKcs and the TLR9 pathway in CpG-ODN signaling is largely unknown. Additionally, since the cytokine response to CpG-ODN is a combination of CpG-ODN-triggered and autocrine- and/or paracrine-mediated immune responses, this response at an early stage of CpG-ODN stimulation may be different from that at a late stagy of stimulation.

In this study, we set out to examine the IL-6 and IL-12 secretion from DNA-PKcs-, TLR9- and DNA-PKcs/TLR9-double deficient DCs in response to different doses of CpG-ODN at different time points. Also, we examined the activation of IKK, IκBα, NF-κB and JNK in these deficient DCs in response to CpG-ODN. Finally, we determined if CpG-ODN can bind to DNA-PKcs in DCs, and whether there is a crosstalk between DNA-PKcs and the TLR9 pathway in CpG-ODN signaling.

## Materials and Methods

### Mice and Bone Marrow-derived Dendritic Cells

*WT*, *DNA-PKcs^−/−^*, *TLR9^−/−^* and *DNA-PKcs^−/−/^TLR9^−/−^* mice were on the C57/B6/129 genetic background, and were bred at University of Hawaii Cancer Center, University of Hawaii, Honolulu, Hawaii, USA. All animal work was approved by the IACUC at University of Hawaii. Bone marrows isolated from each mouse were seeded in a 15-cm petri dish with 15 ml of complete RPMI1640 medium [RPMI1640 supplemented with 1× antibiotics/anti-mycotics (HyClone, Utah), 1× non-essential amino acids (Hyclone), 1 µl/L 2-mercaptoethanol (Sigma, MO) and 10% USA certified, heat-inactive fetal bovine serum (FBS, Invitrogen, CA)] and differentiated into DCs with granulocyte/macrophage-colony stimulatory factor (GM-CSF, Biolegend (CA), 20 ng/ml) for 6.5 or 7.5 days. On day 2 and day 4, 5 ml of fresh RPMI1640 complete medium containing GM-CSF (20 ng/ml) were added, respectively. On day 6.5 or 7.5, DCs in culture medium were collected, and dishes were gently rinsed three times with 1× phosphate buffered saline (PBS). DCs in both medium and rinsed PBS were combined and called suspension DCs. Adhesion DCs were trypsinized and then collected.

### CpG-ODNs and Antibodies

Endotoxin-free CpG-ODN 1018 (5′-TGACTGTGAACGTTCGAGATGA-3′) was synthesized with a phosphorothioate backbone at Trilinker Biotechnology (CA). CpG-ODN 1018-biotin was purchased from Sigma or Trilinker Biotechnology. Streptavidin-agarose beads were from Invitrogen.

Anti-phospho antibodies (Abs) raised against IκBα, IKKα/β, NF-κB and JNK were purchased from Cell Signaling (MA). Anti-TLR9 mouse monoclonal (mAb) was from Sigma; anti-DNA-PKcs mAb was from Becton Dickinson (BD) PharMingen (CA) or Neomarker (CA), and anti-DNA-PKcs polyclonal rabbit antibody was from Santa Cruz Biotech (CA); anti-TRAF6 Ab was from Santa Cruz Biotech; and Alexa fluorescence-conjugated secondary Abs were from Invitrogen.

### ELISA Analysis

Adhesion or suspension DCs were harvested on day 6.5 and then seeded in 96-well plates at 1–2.5×10^5^/well in triplicates. The cells were treated with CpG-ODN, LPS (Invivogen) or R848 (GLS Synthesis, MA) for 6, 12 or 24 hours. The supernatants were collected and the production of IL-6 or IL-12p40 was determined by enzyme-linked immunosorbent assay (ELISA) kits (Biolegend) based on the manufacturer’s instruction.

### Flow Cytometry

0.5–1×10^6^ DCs were stained with APC-conjugated CD 11b, FITC-conjugated MHC-II-I-A/I-E and PE-conjugated CD 11c (Biolegend or BD PharMingen) in PBS plus 1% BSA and washed 3 times with PBS+1% BSA, then analyzed on a FACSCalibur (BD, CA).

### Immunoblotting, Pull-down and Immunoprecipitation Assays

Both adhesion and suspension DCs were harvested and combined, and then washed with PBS twice. The cells were starved for 2 hours in pre-warmed serum-free medium (SFM) (RPMI-1640) and then treated with CpG-ODN (5 µg/ml) in pre-warmed SFM for the indicated durations or left untreated. Whole cell lysates (WCL) were prepared in the lysis buffer I [(150 mM NaCl, 50 mM Tris-Cl (pH7.5), 1 mM EDTA, 1% Triton-X100, 10 mM β-glycerophosphate, 1 mM Na3VO4, and 1× protease inhibitor cocktail (Roche, IN)] and 40–60 µg proteins were separated on a 10% SDS-PAGE and transferred onto PVDF membranes. The membranes were blotted with correspondent primary Abs and secondary Abs-conjugated with HRP, and detected by enhanced chemiluminescence (ECL, Thermo-Piers, IL).

For immunoprecipitation (IP) assays, starved DCs were treated with CpG-ODN (5 µg/ml) in pre-warmed SFM for the indicated durations or left untreated, and then lysed with the lysis buffer I. WCL (200–400 µg) were pre-cleared with 40 µl of protein A and protein G (protein A/G) Sepharose (beads) (1∶1) for 30 min. Pre-cleared WCL were incubated with anti-DNA-PKcs Ab (0.8 µg) at 4°C overnight with rotation, and then incubated with 40 µl of protein A/G beads for another 2 hours. After washing four times with the lysis buffer I containing 1 mM phenylmethylsulfonylfluoride (PMSF, Sigma), proteins with beads were boiled, loaded on 10% SDS-PAGE and transferred onto PVDF membranes followed by immunoblotting (IB) analysis.

For pull-down assays, DCs were treated with CpG-biotin (5 µg/ml) for 0 or 30′ and then washed twice with 1× cold PBS. Cells were lysed with the lysis buffer II [1% digitonin (Sigma), 25 mM HEPES, 100 mM NaCl, 10 mM CaCl2, 5 mM MgCl2, pH7.6] [9] containing 1× protease inhibitor cocktail]. WCL (400 µg) were pre-cleared with protein A/G beads, incubated with 22 µg CpG-biotin for 2 hours, and then with 40 µl streptavidin beads overnight. The beads were washed four times with the lysis buffer II containing 1 mM PMSF, boiled, separated on 10% SDS-PAGE, and transferred onto PVDF membranes followed by IB analysis.

### Immunofluorescence Staining

DCs were seeded in 8-well chamber slides at 1×10^5^/chamber and cultured with complete RPMI-1640 medium overnight. The following day cells were starved for additional 2 hours in pre-warmed SFM and then treated with CpG-ODN (5 µg/ml). Cells were fixed with 4% paraformaldehyde, permeabilized with 0.2% Triton X-100, blocked with 5% BSA in PBS, and stained with primary anti-DNA-PKcs and anti-TRAF6 Abs, and the Alexa fluorescence-conjugated secondary Abs (Alexa 488 or 594). Slides were mounted with anti-fade mounting solution (Electron Microscopy Sciences, PA). The cells were observed under an IX81 Olympus microscope with 60× oil immersion objective powered by 1.6× magnification. The images were recorded by an ORCA R2 CCD mono camera and analyzed by the Metamorph advanced for imaging software.

## Results

### DNA-PKcs is Involved in the IL-6 and IL-12 Response to CpG-ODN in Dose and Time Dependent Manners

**Figure 1:**
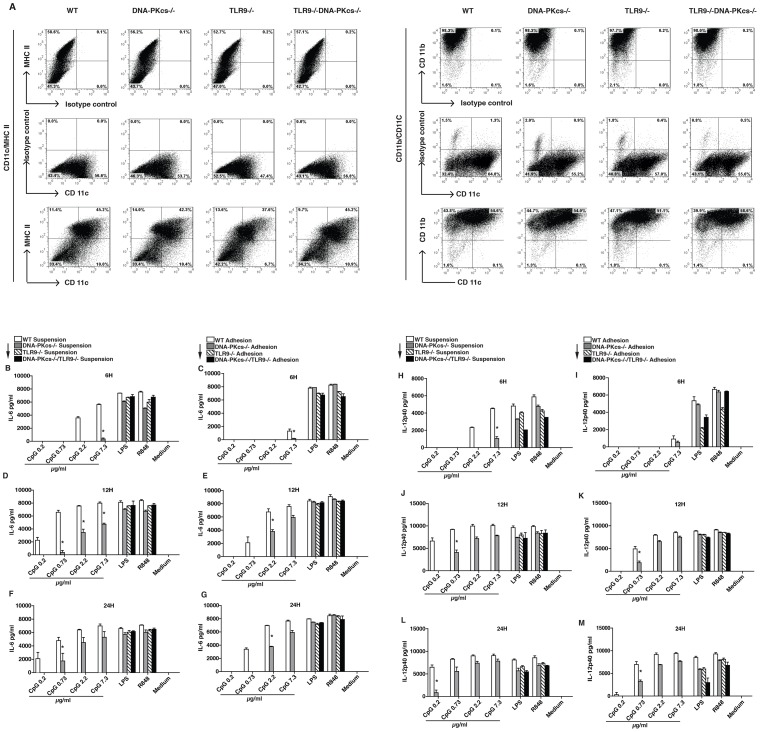
DNA-PKcs is involved in the IL-6 and IL-12 response to CpG-ODN. Bone marrow-derived dendritic cells (DCs) from WT, DNA-PKcs^−/−^, TLR9^−/−^ and DNA-PKcs^−/−/^TLR9^−/−^ mice were harvested based on their natural status: adhesion and suspension. (**A**). Combined DCs (adhesion and suspension) at day 7.5 were subjected to flow cytometry. The levels of CD11b, CD11c and MHC-II expression on DCs were determined. (**B–M**). Adhesion or suspension DCs at day 6.5 were seeded in 96-well plates at 1×10^5^/well in triplicate, and then treated with CpG-ODN (CpG, 0.2, 0.73, 2.2 or 7.3 µg/ml), LPS (0.1 µg/ml), R848 (0.125 µg/ml), or left untreated for indicated time durations. The levels of IL-6 (**B–G**) and IL-12p40 (**H–M**) were determined by ELISA using IL-6 or IL-12p40 ELISA kits. Similar results were obtained in at least 3 independent experiments. Note: bars represent the average of triplicates ± SD. *p<0.05 versus WT groups.

Bone marrows (BM)s of mice can be differentiated into DCs in the presence of granulocyte/macrophage colony-stimulating factor (GM-CSF). Usually, an optimal culture period to generate bone marrow-derived DCs (BMDCs) with GM-CSF is 6–8 days [10]. Thus, we differentiated BMs of WT, DNA-PKcs^−/−^, TLR9^−/−^ and DNA-PKcs^−/−/^TLR9^−/−^ mice with GM-CSF for 7 (6.5 to 7.5) days. All BMs were able to differentiate into DCs, which were present as forms of suspension and adhesion in culture. At 7.5 days, a mixture of adhesion and suspension DCs expressed CD11b, CD11c and MHC-II; CD11C/MHC-II double positive DCs were around 42% and CD11b/CD11c double positive cells were around 55% (Fig. 1A). These results are consistent with a previous methodology study [10]. We also examined percentages of CD11b/CD11c double positive DCs in either adhesion or suspension DCs. The percentages of CD11b/11C double positive DCs in suspension cells were higher (about 56%) than that in adhesion cells (about 40%), and were about 52% for a mixture of both adhesion and suspension cells (Fig. S1).

Next, we determined if suspension DCs are different from that of adhesion DCs in the IL-6 and IL-12p40 response to CpG-ODN, and if DNA-PKs regulates this response by performing CpG-ODN dose response and kinetic assays. As shown in Figs. 1B and 1C, at 6- but not 12- or 24-hours points the IL-6 response to CpG-ODN in suspension DCs was better than that in adhesion DCs. Intriguingly, at the 6-hour point both adhesion and suspension DNA-PKcs-deficient DCs exhibited a clear defect in the IL-6 response to CpG-ODN (Figs. 1B and 1C); at the 12-hour point, both DNA-PKcs-deficient DCs showed an apparent defect in the IL-6 response to CpG-ODN at doses of 0.2, 0.73 and 2.3 but not 7.3 µg/ml (Figs. 1D and 1E); and at the 24-hour point, DNA-PKcs-deficient DCs only showed a defect in the IL-6 response to CpG-ODN at doses of 0.2 and 0.73 µg/ml (Figs. 1F and 1G). These results suggest that DNA-PKcs regulates the IL-6 response to CpG-ODN in a time and dose dependent manner. Similarly, DNA-PKcs-deficient DCs exhibited a defect in the IL-12p40 response to CpG-ODN in a time and dose dependent fashion (Figs. 1I to 1M).

As expected, loss of TLR9 or both DNA-PKcs and TLR9 largely abolished the IL-6 and IL-12p40 response to CpG-ODN (Figs. 1B to 1M). As controls, all WT, DNA-PKcs, TLR9- and DNA-PKcs/TLR9-deficient DCs showed similar IL-6 and IL-12p40 responses to LPS and R848 (Figs. 1B and 1M). Taken together, our results suggest that DNA-PKcs is involved in the cytokine response to CpG-ODN in a time and dose dependent manner.

### DNA-PKcs Regulates Phosphorylation of IKK, IκBα, NF-κB and JNK in Response to CpG-ODN

**Figure 2:**
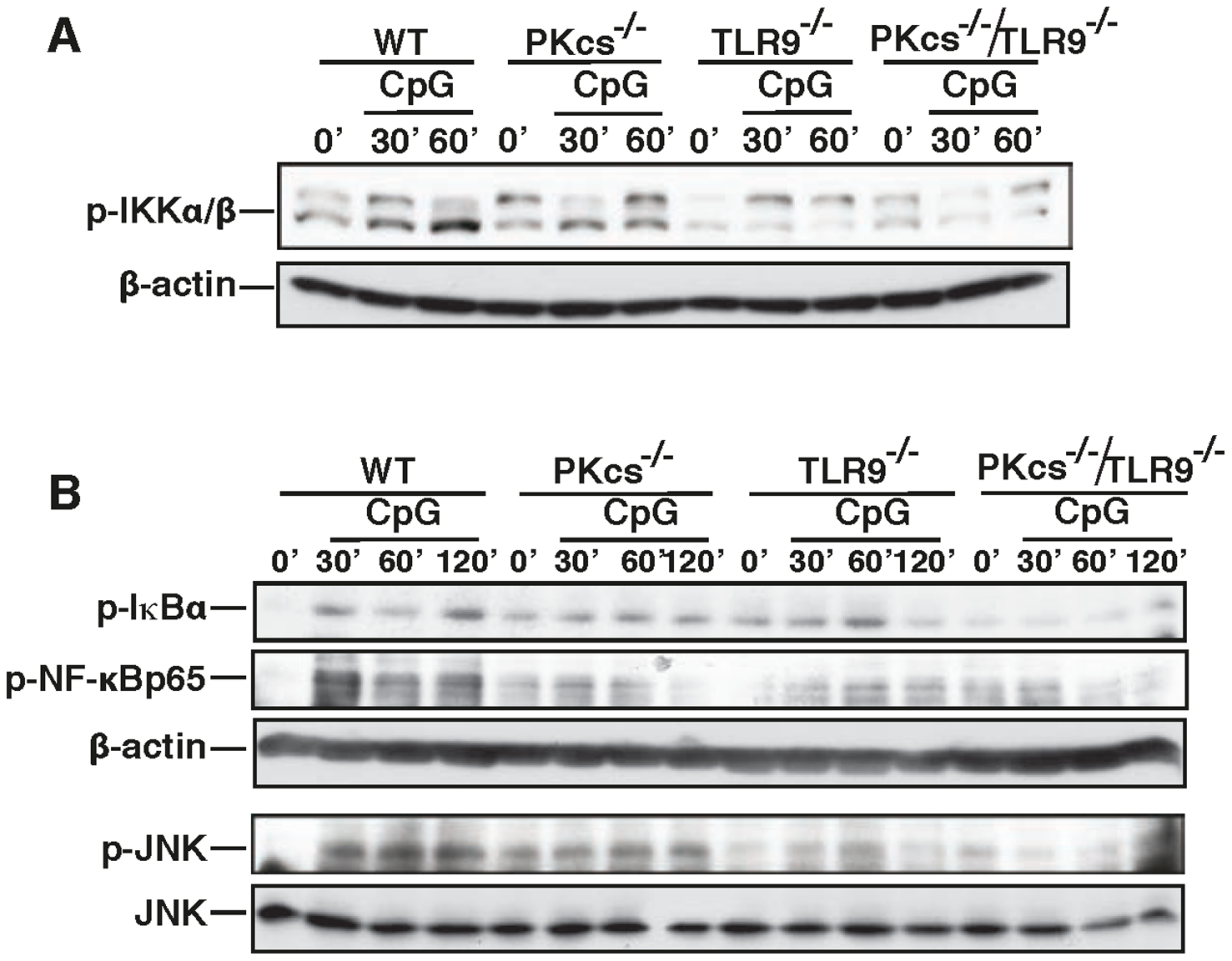
Loss of DNA-PKcs impairs phosphorylation of IKKβ, IκBα, NF-κB and JNK in response to CpG-ODN. (**A**) WT, DNA-PKcs^−/−^ (PKcs^−/−^), TLR9^−/−^ and PKcs^−/−/^TLR9^−/−^ DCs were treated with CpG (5 µg/ml) for the indicated time durations or left untreated. Cell lysates were prepared and phospho-IKKα/β and beta-actin were detected by an immunoblotting (IB) analysis. (**B**) WT, DNA-PKcs^−/−^ (PKcs^−/−^), TLR9^−/−^ and PKcs^−/−/^TLR9^−/−^ DCs were treated with CpG (5 µg/ml) for the indicated time durations or left untreated. The phospho-IκBα, phospho-NF-κB and phospho-JNK as well as JNK and beta-actin were detected. Similar results were obtained from at least three independent experiments.

Because the IKK/NF-κB and JNK/AP-1 pathways are required for pro-inflammatory expression in response to CpG-ODN, we determined if DNA-PKcs regulates these pathways by performing Western blot analysis. As shown in Figs. 2A and 2B, loss of DNA-PKcs reduced the phosphorylation of IKKα/β, NF-κB and JNK as well as IκBα, a substrate of IKK but an inhibitor of NF-κB, in DCs in response to CpG-ODN, As expected, loss of TLR9 also reduced this phosphorylation. Interestingly, loss of both DNA-PKcs and TLR9 further decreased this phosphorylation in response to CpG-ODN.

Taken together, our results suggest that DNA-PKcs regulates the activation of IKK-NF-κB and JNK in CpG-ODN signaling.

### CpG-ODN Binds to DNA-PKcs and TLR9, and Induces the Association of DNA-PKcs with TRAF6

**Figure 3:**
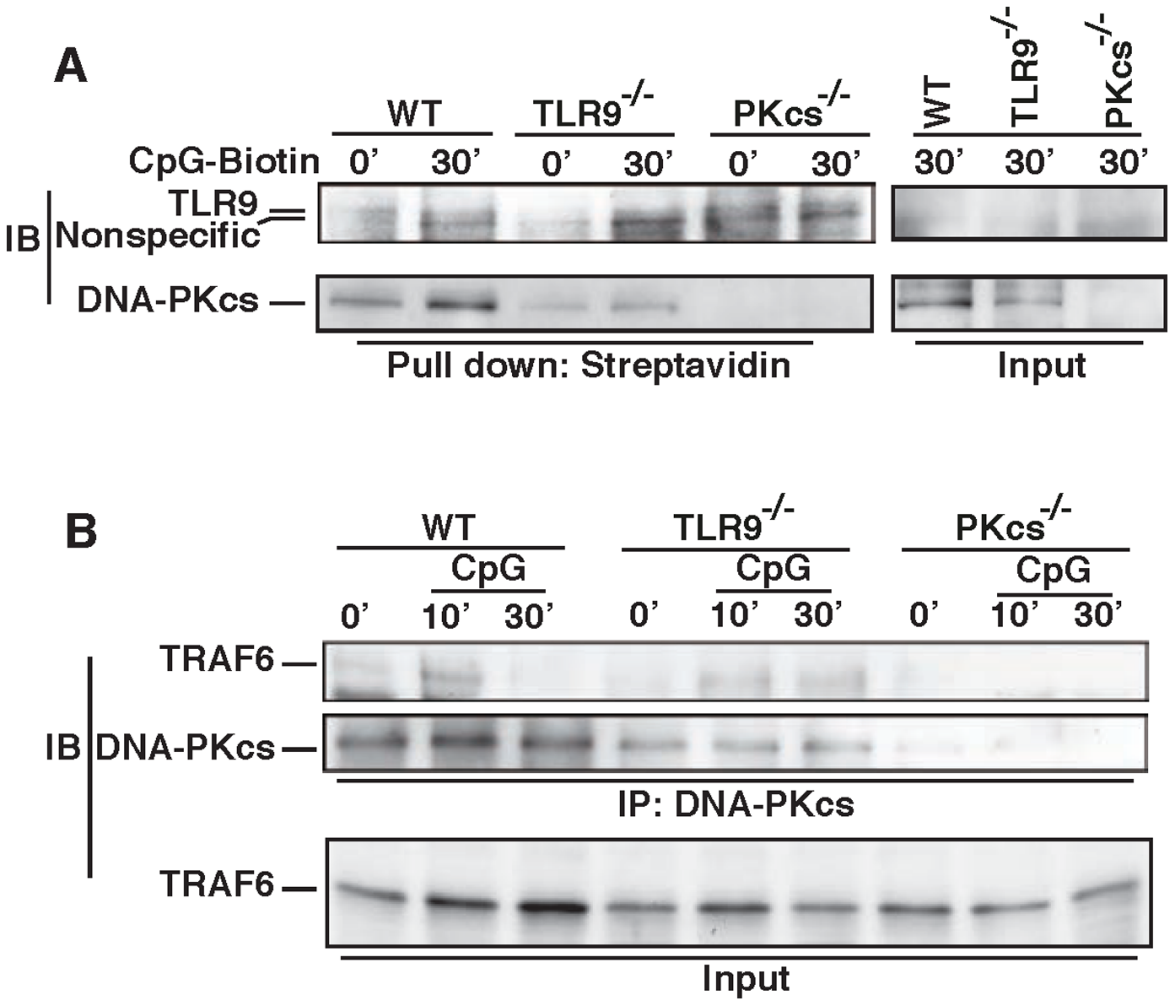
CpG-ODN binds to DNA-PKcs, and induces its interaction with TRAF6. (**A**) CpG-ODN binds to DNA-PKcs and TLR9 in DCs. WT and TLR9^−/−^ DCs were treated with CpG-ODN-biotin (CpG-biotin, 5 µg/ml) for the indicated time durations. Whole cell lystates were prepared with the lysis buffer II, and 400 µg proteins were pre-cleared with protein A/G beads, incubated with 22 µg of CpG-biotin for 2 hours, and then incubated with 40 µl of streptavidin-agarose beads at 4°C overnight. The agarose beads were washed four times with the lysis buffer II. The samples were boiled, loaded onto a 10% SDS-PAGE, and transferred onto a PVDF membrane, which was then blotted with an anti-TLR9 Ab or an anti-DNA-PKcs Ab. Similar results were obtained from three independent experiments. (**B**) CpG-ODN induced DNA-PKcs’s association with TRAF6. WT and TLR9^−/−^ cells were treated with CpG (5 µg/ml) for the indicated time durations. Pre-cleared whole cell lysates (400 µg) were incubated with anti-DNA-PKcs Ab at 4°C overnight and then with 40 µl of protein A/G beads for another 2 hours. Beads were washed four times with the lysis buffer I, boiled, loaded onto a 10% SDS-PAGE and transferred onto a PVDF membrane followed by an IB assay using an anti-TRAF6 Ab or an anti-DNA-PKcs Ab. Similar results were obtained from at least three independent experiments.

To delineate how DNA-PKcs is involved in the activation of the CpG-ODN/pro-inflammatory cytokine pathway, we first determined if CpG-ODN is able to bind to DNA-PKcs in cells. DCs were treated with CpG-ODN-biotin, and whole cell lysates were made and then further incubated with CpG-ODN-biotin (CpG-biotin). As shown in Fig. 3A, CpG-biotin pulled down DNA-PKcs in either treated or untreated WT, TLR9-, but not DNA-PKcs-deficient DCs indicating that CpG-ODN is able to bind to DNA-PKcs. However, more DNA-PKcs was pulled down from CpG-biotin-treated WT or TLR9-deficient DCs indicating that this extra CpG-biotin-DNA-PKcs complex could be formed during the CpG-biotin treatment. As expected, CpG-biotin also bound to TLR9 in WT, DNA-PKcs- but not TLR9-deficient DCs (Fig. 3A). The association of CpG-ODN with DNA-PKcs or TLR9 was further evaluated in DCs upon CpG-ODN-Cy5 stimulation using a fluorescence microscopy. As shown in Fig. S2, CpG-ODN-Cy5 was able to co-localize with DNA-PKcs or TLR9, and in some vesicles three of them were co-localized.

Next, we determined if DNA-PKcs interacts with a relay molecule in the TLR9 pathway. TRAF6 is a key downstream component in the TLR9 pathway and is essential for the innate immune response to CpG-ODN [2]. Because both DNA-PKcs and TRAF6 reside in the cytoplasm, we examined the possibility of their interaction in response to CpG-ODN. In quiescent WT and TLR9-deficient DCs, there was a basal level of the association of DNA-PKcs with TRAF6 (Fig. 3B). Upon stimulation with CpG-ODN this association was further induced, peaked around 10 minutes (min) and decreased after 30 min in WT cells. Unexpectedly, loss of TLR9 did not largely blockade this association indicating that an unidentified but TLR9-independent pathway is responsible for this association.

**Figure 4:**
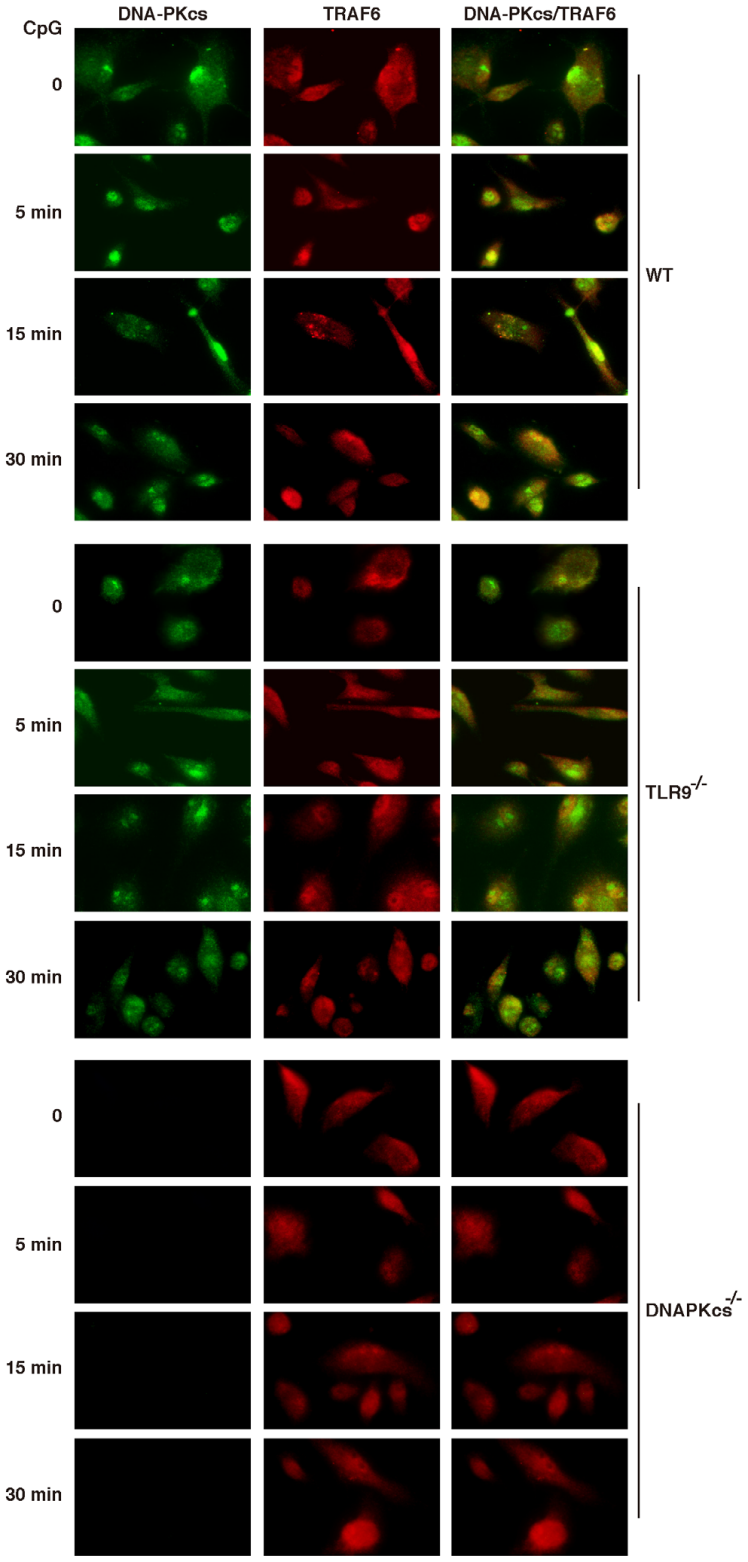
CpG-ODN induces the co-localization of DNA-PKcs with TRAF6. WT, DNA-PKcs^−/−^ and TLR9^−/−^ DCs were seeded in an 8-well chamber slide, and then treated with CpG (5 µg/ml) for the indicated time points. The cells were fixed, permeabilized and immunostained with anti-DNA-PKcs Ab (primary)/Alexa 488 (2^nd^ Ab) (green) and anti-TRAF6 Ab (primary)/Alexa 594 (2^nd^ Ab) (red). Yellow color indicates co-localization of DNA-PKcs and TRAF6. The cells were observed under an IX81 Olympus microscope with 60× oil objective powered by 1.6× magnification. The images were recorded by an ORCA R2 CCD mono camera and analyzed by the Metamorph advanced for imaging software. Similar results were obtained from at least three independent experiments.

To further corroborate the above observation, we performed an immuno-fluorescence assay. Similar to the results presented in Fig. 3B, there was a basal level of co-localization between DNA-PKcs and TRAF6 in both WT and TLR9-deficient DCs, and CpG-ODN further transiently induced this co-localization (Fig. 4). Loss of TLR9 did not largely impair this co-localization.

Taken together, our data suggest that CpG-ODN binds to DNA-PKcs and TLR9, and induces the interaction and co-localization of DNA-PKcs with TRAF6.

## Discussion

We demonstrated that CpG-ODN bound to DNA-PKcs and induced its association with TRAF6, a key relay molecule that transduces signals from TLR9 and MyD88 to its downstream effectors IKK, NF-κB and JNK leading to expression of pro-inflammatory cytokines. We showed that loss of DNA-PKcs impaired IKK, IκBα, NF-κB and JNK phosphorylation, and that DNA-PKcs-deficient DCs exhibited defective IL-6 and IL-12 responses to CpG-ODN in a time- and dose- dependent fashion.

DNA-PKcs is a DNA binding protein and can be activated by CpG-ODN *in vitro* and *in vivo* [3], [11]. CpG-ODN was reported to associate with DNA-PKcs in macrophages if CpG-ODN was pre-packed with liposome containing both DOTAP and DOPE, which helps CpG-ODN escape the endosome [8]. In our experimental conditions CpG-biotin not only pulled down TLR9 in the absence of DNA-PKcs, but it also precipitated DNA-PKcs in DCs in the absence of TLR9. Interestingly, all CpG-ODN, DNA-PKcs and TLR9 could co-localize in some vesicles suggesting that DNA-PKcs could be part of the CpG/TLR9 pathway or DNA-PKcs could interact with the TLR9 pathway upon stimulation with CpG-ODN.

DNA-PKcs has been reported to be involved in the innate immune response to CpG-ODN. DNA-PKcs was able to phosphorylate interferon regulatory factor 3, which is critical for type I IFN expression in response to viral infection [12]. Moreover, DNA-PKcs was shown to be involved in the type I IFN response to CpG-ODN in DCs via a MyD88/IRF7 dependent manner [11]. Recently, it was suggested that SCID macrophages exhibited a defect in ERK activation and IL-10 production, but an enhanced effect on IL-12 production in response to CpG-ODN [8]. On the other hand, two studies indicated that DNA-PKcs was not required for IL-12 expression in DCs 24 or 48 hours after CpG-ODN stimulation at 1 or 3 µM [13], [14]. However, these two observations could not help to explain why CpG-ODN should bind to and activate DNA-PKcs in addition to TLR9 in DCs and macrophages. Because CpG-ODN also induced other pro-inflammatory cytokines, which might synergize with CpG-ODN to further stimulate IL-6 and IL-12 secretion, no apparent defect observed in IL-12 secretion from DNA-PKcs-deficient DCs after long-term CpG-ODN incubations in those studies might not actually reflect the role of DNA-PKcs in CpG-ODN signaling. Additionally, high doses of CpG-ODN might lead to a bypass effect if DNA-PKcs is not an essential component in the CpG-ODN/pro-inflammatory cytokine pathway. Our dose response and kinetic experimental evidence revealed that at an early time point, loss of DNA-PKcs clearly impaired the IL-6 and IL-12 response to CpG-ODN. At late time points, loss of DNA-PKcs only reduced the IL-6 and IL-12 response to CpG-ODN at low doses (0.03 to 0.1 µM = 0.2 µg/ml to 0.73 µg/ml). Our data suggest that DNA-PKcs is a regulator in CpG-ODN signaling and this regulation could play an optimal role in the initial immune response to CpG-ODN, especially when it is at a low concentration.

The molecular mechanism underlying the involvement of DNA-PKcs in the activation of the CpG-ODN/pro-inflammatory cytokine pathway is less understood. It was previously suggested that DNA-PKcs activation by CpG-ODN was abolished in MyD88-deficient DCs indicating that DNA-PKCs is a downstream event of MyD88 [11]. Another study indicated that a point mutation in DNA-PKcs abolished ERK activation by CpG-ODN in SCID macrophages [8]. However, the relationship between DNA-PKcs and the CpG-ODN/TLR9 pathway is still largely unknown.

Previous studies have suggested that TRAF6 was able to mediate the activation of CD40 signaling [15], [16]. Interestingly, a study indicated that CD40 was able to associate with Ku antigens to activate B cells [17]. These studies suggest a possibility that Ku antigens or their complex may associate with TRAF6. Because Ku antigens are the regulatory subunits of DNA-PK, and because TRAF6 is a critical component in the CpG-ODN/TLR9/MyD88 pathway, we thus examined the possibility of the association of TRAF6 with DNA-PKcs in the cytoplasm of DCs in response to CpG-ODN. Our immunoprecipitation results showed that CpG-ODN induced the interaction of DNA-PKcs with TRAF6 in DCs and this interaction was not largely TLR9-dependent because loss of TLR9 only led to a small decrease in this association. Thus, our finding not only provides an insight into the relationship between DNA-PKcs and the TLR9 pathway in which DNA-PKcs interacts with TRAF6, but also offers a mechanism of how DNA-PKcs regulates the IL-6 and IL-12 response to CpG-ODN.

We previously reported that CpG-ODN induces the nuclear translocation of DNA-PKcs in bone marrow-derived macrophages [3]. Consistent with this report, we found that CpG-ODN also induced the nuclear translocation of DNA-PKcs in DCs. This translocation seems to be TLR9-independent because it was not obviously affected by the absence of TLR9. We further observed that, in agreement with above immunoprecipitation data, CpG-ODN induced the co-localization of DNA-PKcs with TRAF6. This co-localization was transient and less dependent on TLR9 indicating that the residual association of TRAF6 with DNA-PKcs may contribute to activation of a TLR9-independent pathway. This pathway could be important or trivial in the pro-inflammatory cytokine response to CpG-ODN under different circumstances.

It is known that TRAF6-mediated activation of the IKK/IκBα/NF-κB pathway is vital for the pro-inflammatory cytokine response to CpG-ODN [18]. Phosphorylation of IKKα/β, IκBα, NF-κB and JNK was impaired in DNA-PKcs- or TLR9- deficient DCs and was further impaired but not totally abolished in DNA-PKcs/TLR9 double deficient DCs. This observation further suggests that DNA-PKcs may not be an absolutely downstream event of TLR9, and that there is a TLR9- or DNA-PKcs-independent pathway in CpG-ODN signaling.

Finally, both DNA-PKcs and TLR9 have been implicated in the development of autoimmune diseases such as lupus. However, studies suggest that the loss of TLR9 reduced production of anti-DNA antibody [19], but exacerbated the disease [20]. Because DNA-PKcs is an auto-antigen and regulates the immune response to CpG-ODN, it is very possible that DNA-PKcs works together with TLR9 or has an independent function in response to DNA/auto-antigen/antibody complexes during the pathogenic process of autoimmune disorders.

In summary, we identified that CpG-ODN was able to bind to DNA-PKcs and induced the interaction of DNA-PKcs with TRAF6. DNA-PKcs regulated the activation of the IKK/NF-κB and JNK pathways and triggered the IL-6 and IL-12 response to CpG-ODN in a time- and dose- dependent manner.

## Supporting Information

Figure S1 **Adhesion DCs (A), suspension DCs (B) or combined DCs (C, adhesion and suspension) at day 7.5 were subjected to flow cytometry.** The levels of CD11b, CD11c and MHC-II on DCs were determined.
(PDF)

Figure S2 **Co-localization of CpG-Cy5 with DNA-PKcs and TLR9 in WT DCs.** WT DCs were treated with CpG-ODN-Cy5 (CpG-Cy5, 1 µM) for 0 or 15 min. The cells were fixed, permeabilized and immunostained with anti-DNA-PKcs Ab (primary)/Alexa 488 (2^nd^ Ab) (green) and anti-TLR9 Ab (primary)/Alexa 350 (2^nd^ Ab) (blue). The yellow or orange colors indicate the co-localization of CpG-Cy5 with DNA-PKcs; the pink color indicates the co-localization of CpG-Cy5 with TLR9; the rainbow or white color indicates the co-localization of CpG-Cy5 with DNA-PKcs and TLR9. The cells were observed under an IX81 Olympus microscope with 60× oil objective powered by 1.6× magnification. The images were recorded by an ORCA R2 CCD mono camera and analyzed by the Metamorph advanced for imaging software. As controls, DNA-PKcs- or TLR9-deficient DCs were also respectively stained with anti-DNA-PKcs or anti-TLR9 antibodies.
(TIFF)
