# Supplementary figures and images for: Involvement of DNA-PKcs in the IL-6 and IL-12 Response to CpG-ODN Is Mediated by Its Interaction with TRAF6 in Dendritic Cells
Source: PLoS One. 2013 Mar 22;8(3):e58072. doi: 10.1371/journal.pone.0058072 (PMC3606245; doi:10.1371/journal.pone.0058072)

Figure S1

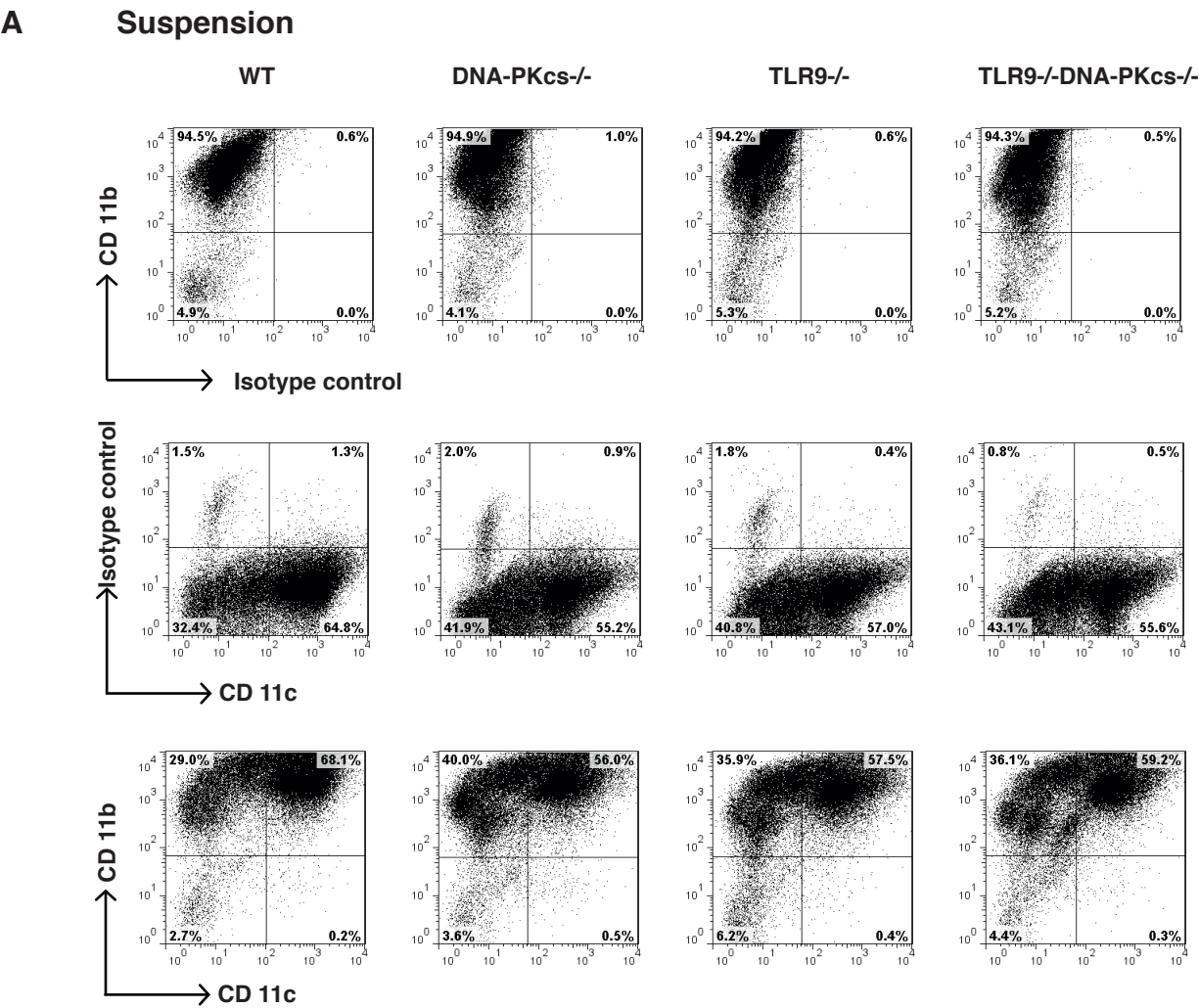

Figure S1

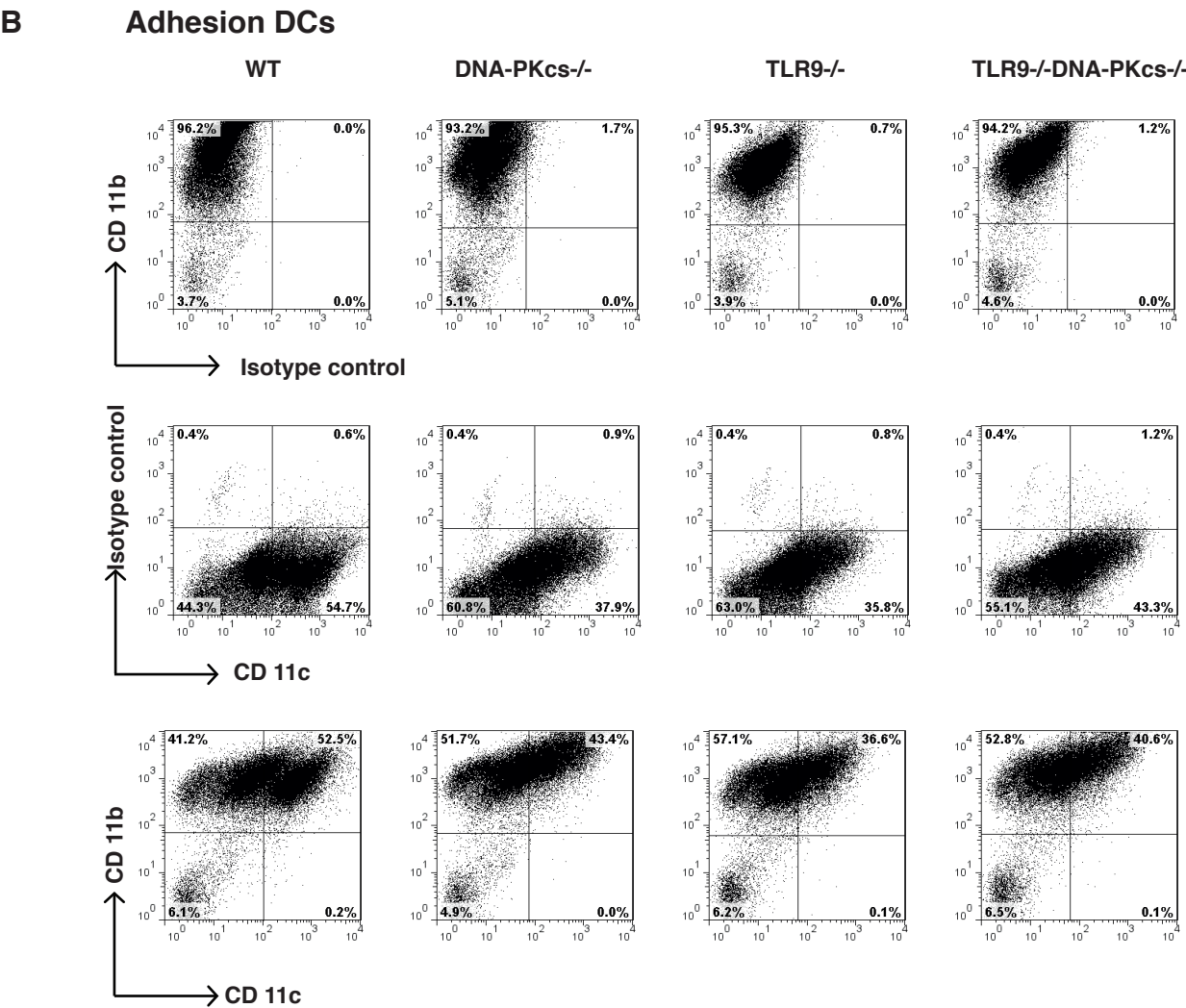

**C Combined with adhesion and suspension DCs**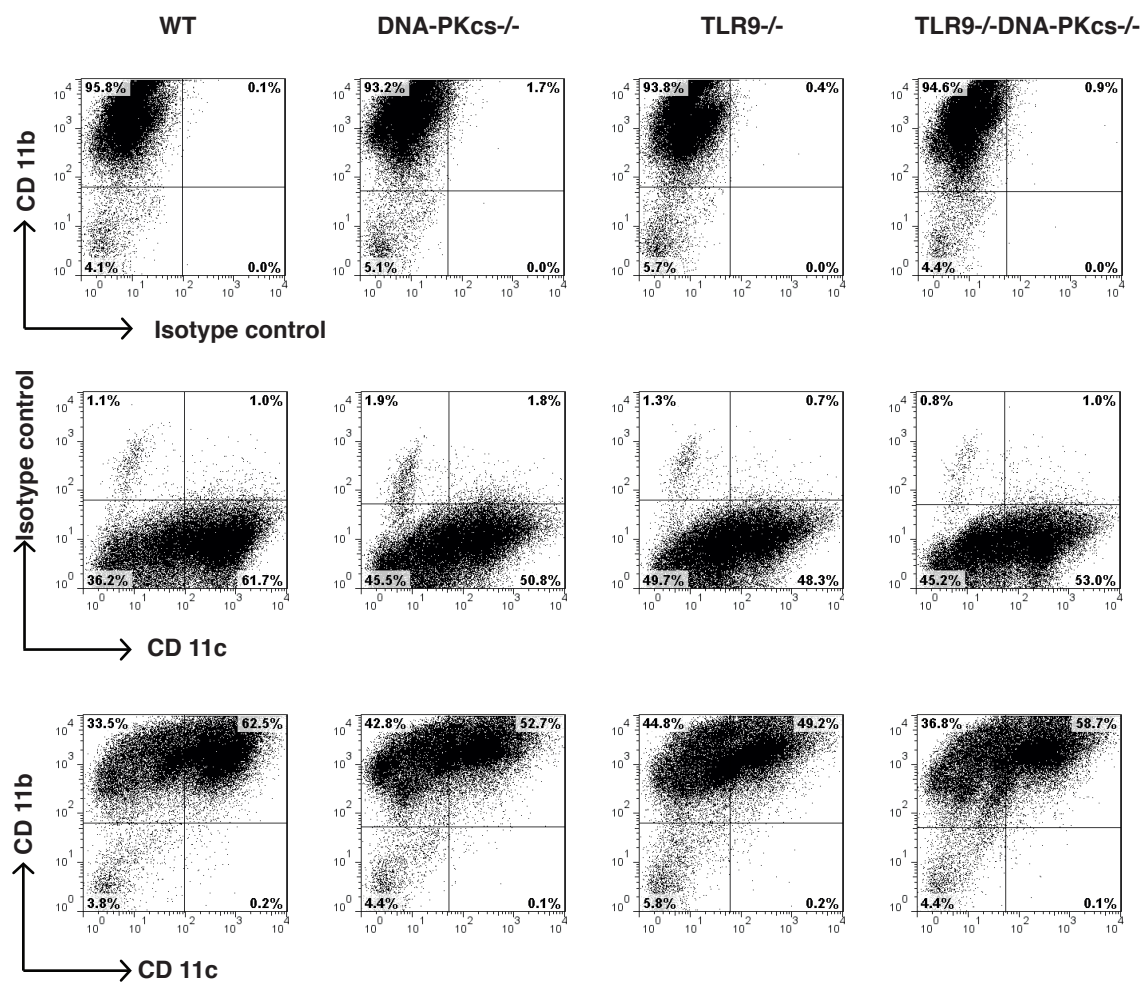

Supplement: Figure S1 — Adhesion DCs (A), suspension DCs (B) or combined DCs (C, adhesion and suspension) at day 7.5 were subjected to flow cytometry. The levels of CD11b, CD11c and MHC-II on DCs were determined. (PDF) [file pone.0058072.s001.pdf]

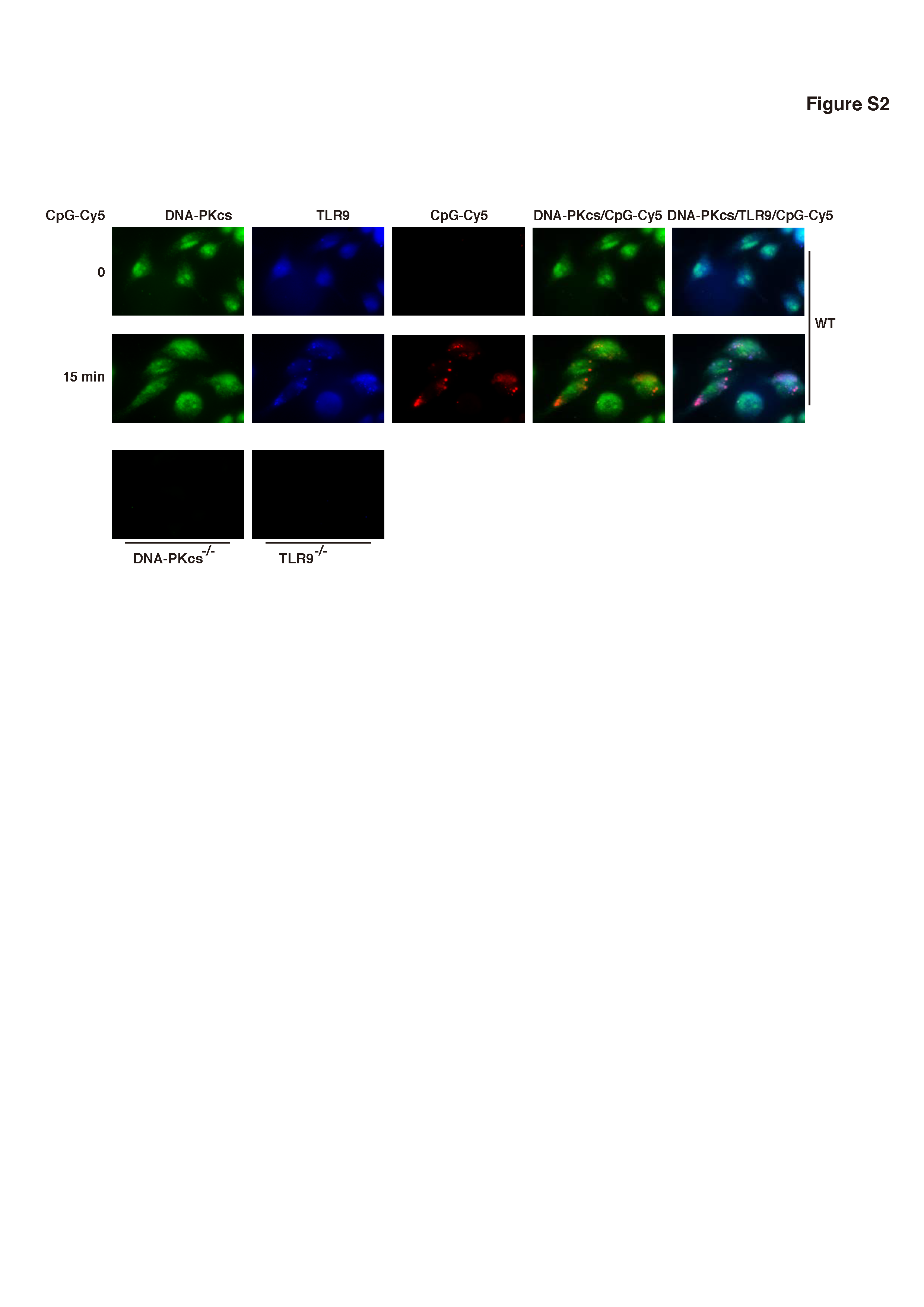

Supplement: Figure S2 — Co-localization of CpG-Cy5 with DNA-PKcs and TLR9 in WT DCs. WT DCs were treated with CpG-ODN-Cy5 (CpG-Cy5, 1 µM) for 0 or 15 min. The cells were fixed, permeabilized and immunostained with anti-DNA-PKcs Ab (primary)/Alexa 488 (2nd Ab) (green) and anti-TLR9 Ab (primary)/Alexa 350 (2nd Ab) (blue). The yellow or orange colors indicate the co-localization of CpG-Cy5 with DNA-PKcs; the pink color indicates the co-localization of CpG-Cy5 with TLR9; the rainbow or white color indicates the co-localization of CpG-Cy5 with DNA-PKcs and TLR9. The cells were observed under an IX81 Olympus microscope with 60× oil objective powered by 1.6× magnification. The images were recorded by an ORCA R2 CCD mono camera and analyzed by the Metamorph advanced for imaging software. As controls, DNA-PKcs- or TLR9-deficient DCs were also respectively stained with anti-DNA-PKcs or anti-TLR9 antibodies. (TIFF) [file pone.0058072.s002.tiff]
